# Vitamin D and Covid-19: an update on evidence and potential therapeutic implications

**DOI:** 10.1186/s12948-020-00139-0

**Published:** 2020-11-19

**Authors:** Giuseppe Murdaca, Giovanni Pioggia, Simone Negrini

**Affiliations:** 1 Department of Internal Medicine, University of Genoa and IRCCS Ospedale Policlinico San Martino, Viale Benedetto XV, n. 6, 16132 Genova, Italy; 2 Institute for Biomedical Research and Innovation (IRIB), National Research Council of Italy (CNR), 98164 Messina, Italy

**Keywords:** Vitamin D, Covid-19

## Abstract

The world is now experiencing its third major epidemic of coronavirus (CoV) infections began in Wuhan, Hubei, China, in late 2019 and named COVID-19. After an initial explosive outbreak of pneumonia of unknown etiology in China, the disease spread first to neighboring Asian countries and then worldwide. Patients with COVID-19 presented with a constellation of symptoms such as fever, dry cough, dyspnea, sore throat, and nasal congestion and radiological findings showed bilateral lung glassy opacities. Vitamin D has many mechanisms by which it reduces the risk of microbial infection and death, including physical barrier, cellular natural immunity, and adaptive immunity. Vitamin D supplementation has shown favorable effects in viral infections including influenza and HIV. The effects of vitamin D supplementation during covid 19 infection remain controversial. Looking ahead, clinical studies are needed to define better cut offs for vitamin D levels and, finally, which dosage is the best.

## Key points

- Vitamin D reduces the risk of microbial infection and death.
- Vitamin D supplementation has shown favorable effects in viral infections including influenza and HIV.
- Vitamin D is a negative endocrine renin-angiotensin system (RAS) modulator.
- Vitamin D increases expression and concentration of ACE2, MasR and Ang-(1–7) and has a potential protective role against acute lung injury/acute respiratory distress syndrome.
- Despite the potential effectiveness of vitamin D as an antiviral, more solid data are needed to support this claim.

## Background

Currently, a major epidemic of coronavirus (CoV) infection is occurring worldwide. The current CoV infection started in Wuhan, Hubei, China, late in 2019 [1]. On February 11, 2020, the World Health Organization (WHO) named the epidemic COVID-19 [2]. In 2002, the first epidemic of a CoV infection also began in China, for which the clinical features included severe acute respiratory syndrome (SARS)-CoV [3], while another—currently ongoing in the Middle East—was first reported in 2012 [4] and is named Middle East respiratory syndrome (MERS)-CoV. The epidemic of COVID-19 is the third one which, starting from an explosive outbreak only in China and subsequently in neighboring Asian countries, spread worldwide [5], with several countries including the USA, Italy, Spain, China, Germany and Iran leading in terms of the most confirmed cases and related deaths [6]. Samples gathered from throat and nasal swabs are useful to perform a polymerase chain reaction (PCR) analysis which is able to detect the SARS-CoV-2 infection [7]. The main symptom of COVID-19 is fever (85% of cases), and in early onset 45% of cases include febrile, dyspnea, dry cough, sore throat, nasal congestion, and radiological findings showing bilateral lung glassy opacities. Damage to the lung tissue may result in acute respiratory distress syndrome (ARDS), of which a potential consequence is septic shock. These are the two major COVID-19 contributors to hospitalization in an intensive care unit (ICU) and mortality in patients who are more than 60 years old. Several other symptoms, such as bone and muscle aches, chills, and headaches are under observation [7]. Minor reported symptoms include nausea or vomiting and diarrhea, respectively in 5% and 3.7% of cases [7]. Furthermore, anosmia and ageusia appear to be frequent clinical features in COVID-19 patients [8]. Several works have reported how the group of subjects who are smokers, especially at an older age, tend to have a higher density of angiotensin converting enzyme 2 (ACE2) receptors [9]. COVID-19 has an incubation period of around 2–14 days, with a mean of 3 days and a fatality rate (CFR) of 12% worldwide [9]. The suggested cut-off for self-quarantine is 14 days [7]. Subjects with COVID-19 show decreased or normal leucocytes and lymphocytopenia, as well as a systemic elevation of pyrogenic cytokines such as interleukin (IL)-6, IL-10, and tumor necrosis factor (TNF)-α [7, 10]. When subjects are in a critical condition, several studies have reported an increase of neutrophilia and elevated D-dimer, as well as urea nitrogen (BUN) and creatinine in the blood plasma [7, 10, 11]. Increased plasma levels of IL-2, IL-7, IL-10, granulocyte colony stimulating factor, 10 kD, interferon (IFN)-γ-induced-protein-10, monocyte chemoattractant protein-1, and macrophage inflammatory protein 1-α have also been reported [10]. Early diagnosis, isolation, and treatment are essential to cure the disease and control the epidemic. Serum antibody detection is of great significance in the diagnosis of infected patients, especially for patients with a negative nucleic acid test. Simultaneous detection of both IgM and IgG antibodies helps to identify the stage of the infection. Generally, the antibody profile against COVID-19 shows a typical IgM and IgG pattern profile. SARS-specific IgM antibodies appear about two weeks after infection, and disappear at the end of week 12, while the IgG antibodies may last for months or even many years [12]. For COVID-19, however, the longitudinal pattern of antibodies remains unclear [13]. Presently, a dedicated treatment has not yet been developed, and trials of antiviral drugs remain experimental. Moreover, an official vaccine has not yet been approved; the completion time for such a vaccine is estimated to be by June 2021 [9]. Taking into account the effects of corticosteroids in prolonging the viral shedding time and in maintaining the systemic anti-inflammatory state while minimizing the precipitation of ARDS, dyspnea, and severe pneumonia, several attempts have been made in the management of such pathologies of viral pneumonia to use systemic corticosteroids. However, their application remains controversial. For this reason, the use of corticosteroids is not recommended outside of clinical trials, or unless otherwise indicated [9]. Notably, heparin treatment can contribute to reduced mortality in patients with severe COVID-19 and sepsis-induced coagulopathy [14]. However, chloroquine (CQ) and its hydroxychloroquine derivative (HCQ) have been administered to patients with severe symptoms [15–18]. It has been reported that anesthetics (i.e., propofol) can disrupt ordered monosialotetrahexosylganglioside1 (GM1) lipid rafts. These same lipid rafts recruit the COVID-19 surface ACE2 to an endocytic entry point, away from phosphatidylinositol 4,5 bisphosphate (PIP2) domains [19]. Of note, HCQ acts through anesthetic-like mechanism disrupting ACE2 localization at both GM1 rafts and PIP2 domains decreasing the ability of the virus to cluster and enter the cell [20]. Furthermore, HCQ seems to inhibit important functional proteins for COVID-19 replication, with potency increasing in the series PLpro, 3CLpro, RdRp [21]. It has been reported that supplements with vitamin A, B, C, D, and E seems to have a beneficial effects in patients with viral infections as COVID-19 [2, 22–25]. Within such a panorama, it is worth mentioning that vitamin D mitigates the scope of acquired immunity and regenerates the endothelial lining. In this review, we discuss the potential role of vitamin D supplementation in COVID-19 infection.

### Vitamin D metabolism

Due to the thermal action of UVB radiation reaching 7-dehydrocholesterol in the skin, vitamin D3 is produced. Following this reaction, vitamin D3 or oral vitamin D is then converted in the liver to 25(OH)D and then, in the kidneys or other organs, to the hormonal metabolite 1,25(OH)2D (calcitriol) [26, 27]. Calcitriol enters the nuclear receptor of vitamin D, binding with DNA. This binding allows a direct interaction with the regulatory sequences near target genes, for which chromatin active complexes genetically and epigenetically contribute to modifying the transcriptional output [27]. Calcitriol contributes to regulating the concentrations of serum calcium through a feedback loop with parathyroid hormone (PTH), and in this way modifies many important functions in the body [26].

### Vitamin D and the host immune response

**Fig. 1:**
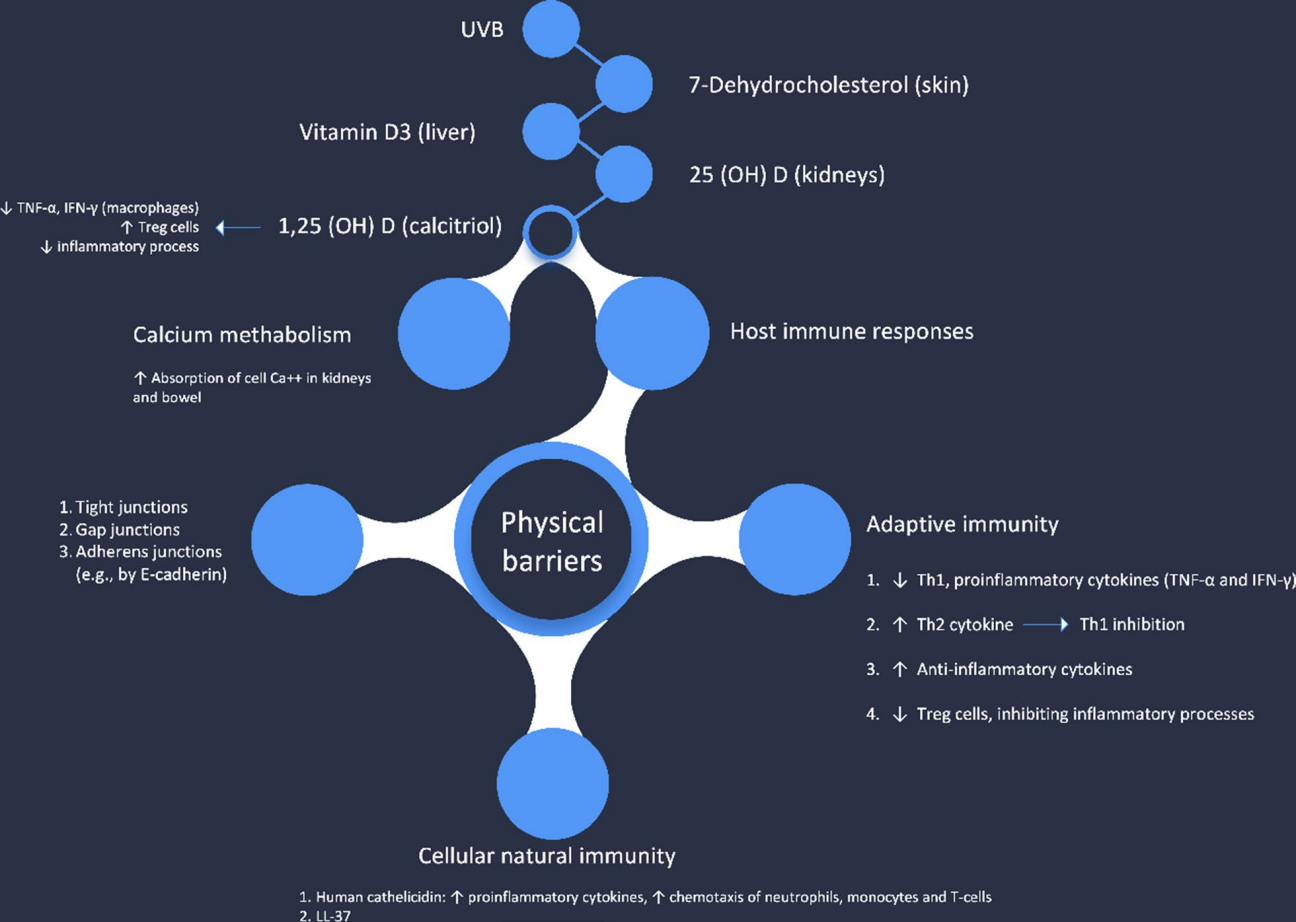
Effects of vitamin D on immune system, calcium metabolism and cytokines

Vitamin D contributes to reducing the risk of microbial infection and death, mainly involving actions grouped into three categories: physical barriers, cellular natural immunity, and adaptive immunity [28]. Innate cellular immunity is strengthened by vitamin D actions partly through the induction of antimicrobial peptides, including the human cathelicidin LL-37 and by 1,25-dihdroxyvitamin D and defensins, while maintaining tight junctions, gap junctions, and adherens junctions [29–32]. In particular, it is worth mentioning the effects of cathelicidins, which exhibit a direct antimicrobial effect versus a wide range of microbes. These include, among others, Gram-positive and Gram-negative bacteria, enveloped and non-enveloped viruses, and fungi [33]. Cathelicidin displays other functions including the induction of a variety of pro-inflammatory cytokines, stimulation of the chemotaxis of neutrophils, monocytes, macrophages, and T lymphocytes into the site of infection, and promotion of the clearance of respiratory pathogens by inducing apoptosis and autophagy of infected epithelial cells [34, 35]. Furthermore, 1,25(OH)2D–vitamin D receptor complex acts on the cathelicidin gene promoter vitamin D response elements to enhance transcription of cathelicidin [36]. COVID-19 subjects show innate behaviour of the immune system in response to viral and bacterial infections, generating both pro-inflammatory and anti-inflammatory cytokines [37]. Vitamin D may contribute to reducing the production of pro-inflammatory T helper (Th)1 cytokines, (TNF-α and IFN-γ), and increases the expression of anti-inflammatory cytokines by macrophages [38, 39]. Vitamin D promotes cytokine production by Th2 lymphocytes, enhancing the indirect suppression of Th1 cells by complementing this with actions mediated by a multitude of cell types [40]. It also favors induction of the T regulatory (Treg) cells, thereby inhibiting inflammatory processes [41, 42]. Serum vitamin D concentrations tend to decrease with age due less time spent in the sun and lower levels of 7-dehydrocholesterol in the skin [43, 44]. Notably, vitamin D concentrations in the serum can be reduced by antiepileptics, antineoplastics, antibiotics, anti-inflammatory agents, antihypertensives, antiretrovirals, endocrine drugs, and some herbal medicines, through the activation of the pregnane-X receptor [45]. The expression of genes related to antioxidation (glutathione reductase and the glutamate–cysteine ligase modifier subunit) is enhanced by supplementation of Vitamin D [46] and, thus, the increased glutathione production spares the use of vitamin C, which has antimicrobial activities [47, 48]. The effects of vitamin D on the immune system are shown in Fig. 1.

### Vitamin D and COVID-19

Winter vitamin D supplementation seems to reduce the risk of developing influenza. Two randomized controlled trials (RCTs) have reported beneficial effects along these lines [49, 50]. A few studies have presented some limitations in the design of these clinical trials; for instance, an RCT in Japan which included many subjects who had been vaccinated against influenza and did not measure baseline vitamin D levels reported no benefit from the administration of vitamin D [51]. However, the two most recent RCTs included participants with above average mean baseline vitamin D concentrations [52, 53]. Gruber-Bzura et al. [54] reported that vitamin D should reduce the risk of influenza, even if other studies are needed to confirm these findings. Furthermore, the potential beneficial effects of vitamin D supplementation have also been described in human immunodeficiency virus-1 (HIV) infection. Indeed, Mansueto et al. [55] reported that preclinical experiments have demonstrated that treatment of peripheral blood mononuclear cells with 1,25(OH)2D decreased the cell susceptibility to HIV infection by inhibiting viral entry, modulating the expression of CD4 + cell surface antigens, damping viral p24 production, and limiting monocyte proliferation. Baseline vitamin D levels lower than 32 ng/mL were independently associated with progression to a more advanced HIV stage. These findings seem to confirm the potential benefits of the administration of vitamin D in HIV patients, even if assay variability and costs, lack of a clear target range, absence of proven supplementation benefits, confounding from osteoporosis and older age, limited RCT data in HIV-infected patients, and finally the inability to distinguish the effects of vitamin D prevent routine screening of vitamin D levels. In terms of the potential impact of vitamin D supplementation in patients with COVID-19 infection, experimental reports have shown vitamin D has a role in reducing the risk of COVID-19, including consideration of the fact that the outbreak occurred in winter (a time when serum vitamin D levels are lowest), and the fact that vitamin D deficiency contributes to acute respiratory distress syndrome and case-fatality rates increasing with age and with chronic disease comorbidity, both of which are associated with a lower 1,25(OH)2D concentration [2]. However, it is reasonable to hypothesize that vitamin D supplementation may enhance host immune responses against COVID-19 and its aggressive effects on all organ systems. High-dose vitamin D supplementation may be considered for subjects with laboratory confirmed deficiency, particularly the elderly, obese, those with dark skin, and those individuals living at higher latitudes. Thirty-five degrees North also happens to be the latitude above which people do not receive sufficient sunlight to retain adequate vitamin D levels during winter and, thus, vitamin D supplementation is needed. Based on its protective effects in subjects at risk of chronic diseases, including cancers, cardiovascular disease (CVD), respiratory tract infections, diabetes mellitus, and hypertension, it can be assumed that vitamin D supplementation and the associated increase of serum vitamin D levels above 50 ng/ml (125 nmol/l) may have beneficial effects in reducing the incidence and severity of various viral diseases, including COVID-19 [5, 56]. Given the well-known deleterious consequences of malnutrition [57], and keeping in mind the peculiarities of the ICU setting, Caccialanza et al. [58] planned a pragmatic protocol for early nutritional supplementation of non-ICU patients hospitalized for COVID-19. Almost all COVID-19 hospitalized patients present at admission with severe inflammation and anorexia, leading to a major reduction of food intake, and a substantial percentage of them develop respiratory failure requiring non-invasive ventilation (NIV) or continuous positive air-way pressure (CPAP) within a few days. Furthermore, taking measurements of weight and height may be difficult, mainly due to a lack of scales, as well as in consideration of the required hygienic precautions. Moreover, measurements of body composition may not be regularly gathered during the peak of an epidemic, due to the associated safety concerns. Parenteral nutrition (PN) may only partially fit the needs of subjects with pre-ICU COVID-19, because central infusion lines are rarely available outside ICU wards, and as requirements for energy are likely to be elevated considering the concurrent severe acute inflammatory state and that the average BMI of COVID-19 patients is often high upon admission. Tian et al. [59] confirmed gastrointestinal clinical and laboratory features in COVID-19 from case reports and retrospective clinical studies. As previously reported, ACE2 is the receptor which hosts COVID-19 entry into the cells of the intestine and alveoli, with dysregulation of the renin-angiotensin system which contributes to massive cytokine activation; this can be potentially fatal in ARDS. However, vitamin D deficiency may also contribute to airway/gastrointestinal infections. Of note, elderly Italians have a very high prevalence of hypovitaminosis D, with a peak during the winter season [60]. It has been proven that vitamin D in mice attenuates acute lung injury caused by lipopolysaccharide-induction, by blocking the effects of the angiopoietin (Ang)-2-Tie-2 signaling pathway and on the renin-angiotensin pathway [61]. Furthermore, Malek Mahdavi confirmed that vitamin D is a negative endocrine renin-angiotensin system (RAS) modulator and inhibits renin expression and generation. It can induce ACE2/Ang-(1–7)/MasR axis activity and inhibits renin and the ACE/Ang II/AT1R axis, thereby increasing expression and concentration of ACE2, MasR and Ang-(1–7) and having a potential protective role against acute lung injury/ARDS. Therefore, he suggested that vitamnin D may be a potential therapeutic approach to combat COVID-19 and induced ARDS [62, 63]. Although it is more likely that any protective effect of vitamin D against COVID-19 is related to suppression of cytokine response, it seems possible that vitamin D prophylaxis (without over-dosing) may decrease the severity of illness caused by COVID-19, especially in settings where hypovitaminosis D is common [64]. Moreover, Marik et al. [65] suggested that hypovitaminosis D may partly explain the geographic variations in the reported case fatality rate of COVID-19, indicating that supplementation with vitamin D may reduce the mortality from this pandemic. These findings confimed that persistent deficiency in vitamin D level may activate the RAS that induces lung fibrosis [65]. Furthermore, hypovitaminosis D promotes the renin-angiotensin system (RAS), chronic activation of which may lead to chronic CVD and decreased lung function [66]. Tsujino et al. [67] recently reported, both in mouse models of bleomycin-induced interstitial pneumonia and human cells, that vitamin D3 is activated in lung tissue and this activation shows a preventive effect on experimental interstitial pneumonitis. Martineau et al. [68] confirmed the safety and the protective effect against acute respiratory tract infection of regular oral vitamin D2/D3 intake (up to 2000 IU/d without an additional bolus), especially in subjects with vitamin D deficiency. Vitamin D supplementation increases the peripheral CD4 + T lymphocyte count in HIV infection [69], and one of the main manifestations of severe COVID-19 infection is lymphopenia. Hanff et al. [70] speculated that CVD or RAS blockade drugs might augment ACE2 levels, increasing the available substrate for COVID-19 infection. COVID-19 infection is thought to downregulate ACE2 function, leading to toxic angiotensin II over-accumulation, which in turn may contribute to ARDS or fulminant myocarditis. Notably, hypovitaminosis D seems to increase the risk for thrombosis, and vitamin D controls the expression of several genes relevant to cellular proliferation, differentiation, apoptosis, and angiogenesis [71]. The administration of a high dose of 25(OH) vitamin D significantly decrease the need to admit COVID-19 patients to ICU [72]. However, hCAP-18 is the only human cathelicidin hydrolyzed by proteinase 3 between an alanyl and a leucyl residue to produce an antibacterial peptide LL-3 that also inhibits platelet aggregation reducing the risk of thrombus formation. LL-37 can reduce phosphorylation of Src kinase and Akt^Ser473^, decrease platelet spreading on immobilized fibrinogen and inhibit P-selectin expression on platelets [73]. Endothelial cells could be infected by COVID-19 through ACE2 receptors on the endothelium inducing endothelial dysfunction [74]. Induction of endothelial dysfunction may be also relevant to an inadequate degree of 1,25(OH) 2D3, which cannot efficiently act as a ligand for vitamin D receptor (VDR), resulting in the disorder of vitamin D-binding protein binding to the ligand for VDR on the endothelium. Furthermore, TNF-α increases interferon (IFN)-α inducing secondary endothelial dysfunction and, thus, increasing the risk of endothelialitis, coagulopathy and thrombosis. Vitamin D deficiency makes patients more at risk of death [74]. These findings confirm that hypovitaminosis D may be associated with an increased risk of severity in COVID-19 and, thus, are further evidence of the positive role played by vitamin D supplementation in the immune response [64, 75, 76]. Interestingly, Italy and Spain, perhaps contrary to expectation, each have a relatively high prevalence of vitamin D deficiency [77]. Intensive vitamin D supplementation as a possible prophylaxis could be considered in addition to exposure to UVB rays, as we are still lacking specific and effective treatments for COVID-19. The good tolerability and safety of even of high doses of vitamin D makes vitamin D supplementation consistent with the primum non nocere principle. Investigations on vitamin D status and VDR gene polymorphisms could explain the unusual behavior of COVID-19′s spread, and the variety of clinical presentations and outcomes [78]. Given the link between diminished immune function and individuals with obesity, this raises important questions about the possibility for greater viral pathogenicity in this population [79]. Increased adiposity may undermine the pulmonary microenvironment (e.g., alveoli), wherein viral pathogenesis and immune cell trafficking could contribute to a maladaptive cycle of local inflammation and secondary injury, further worsened by the presence of high blood pressure and diabetes mellitus—both of which are typically connected to obesity [80, 81]. In patients with type 2 diabetes mellitus hyperinsulinaemia promotes lowers vitamin D status via sequestration into adipocytes decreasing plasma membrane negative charge between red blood cells, platelets and endothelial cells, and, thus, increasing agglutination and thrombosis [82]. Particular attention must be dedicated to treatment with testosterone; its safety is under discussion due to recent evidence in patients with COVID-19, in particular in hypogonadal men with a greater genetic predisposition, of an increased frequency of venous thromboembolism (VTE)—a clinical element associated with a worse prognosis [83]. However, the risk of VTE in patients treated with testosterone is very current. In a recent case-crossover study, 39,622 men were enrolled and 3110 of them (7.8%) had hypogonadism. Testosterone replacement therapy was associated with a higher risk of VTE in men with (odds ratio 2.32) and without (odds ratio 2.02) hypogonadism [84]. What is the link between the male testosterone levels and the risk of severe lung involvement in patients with COVID-19? Based on the role of the variation in androgen levels throughout life [85], testosterone could play a double-edged role in the natural history of COVID-19 infection. In the early phase, the immunosuppressive action of testosterone could explain male’s greater susceptibility to infection therefore leading to speculate a protective role of ADT. On the contrary when the infection occurred, in elderly males who frequently develop ARDS, late-onset hypogonadism could result in a lower immunosuppressive effect on the cytokine storm [86]. Indeed, in subjects with hypogonadism, testosterone inhibits the immune stimuli–induced secretion of proinflammatory cytokines, such as TNF-α and IFN-γ, which can be measured in the peripheral blood leukocytes, demonstrating a worsening of the systemic inflammatory response [83]. These findings further support the hypothesis that vitamin D prevents the cytokine storm and subsequent ARDS that is commonly the cause of mortality in COVID-19 infection [80, 81]. In subjects with HIV infections, a deficiency of vitamin D is associated with increased levels of IL-6 [87], while in diabetic mice supplementation of vitamin D can reduce excess IL-6 levels [88].

## Conclusion and perspectives

Data reported in the literature concerning the effects of vitamin D supplementation are yet controversial in patients with COVID-19. The pathology of COVID-19 involves a complex interaction between COVID-19 and the immune system. However, vitamin D has multiple immunomodulating actions. Of note, vitamin D favors the ability of macrophages to mature and prevents macrophages from releasing too many inflammatory cytokines and chemokines. Furthermore, Vitamin D supplementation has shown favorable effects in numerous viral infections. However, data still available on the effects of vitamin D supplementation during covid 19 infection remain controversial. Looking ahead, clinical studies are needed to define better cut offs for vitamin D levels and, finally, which dosage is the best.

## Data Availability

Not applicable.
